# Supplementary material for: Mitochondrial Dynamics Markers and Related Signaling Molecules Are Important Regulators of Spermatozoa Number and Functionality
Source: Int J Mol Sci. 2021 May 27;22(11):5693. doi: 10.3390/ijms22115693 (PMC8199422; doi:10.3390/ijms22115693)
Supplement: Supplementary file 1 [file ijms-22-05693-s001.zip › ijms-1200539-SI.pdf]

Manuscript Title
Mitochondrial dynamics markers and related signaling molecules are important regulators of spermatozoa number and functionality

Authors
Isidora M Starovlah, Sava M Radovic Pletikosic, Tatjana S Kostic, Silvana A Andric

SUPPLEMENTARY RESULTS

Mitochondrial membrane potential (Δψ) measurement using TMRE fluorescent dye in spermatozoa 3 hours after *in vivo* adrenaline treatment of adult rats.

Three-months-old male *Wistar* rats were used for the experiment. Animals were divided into two groups, control and adrenaline treated. All the animals were treated with intramuscular injection at 07^{00} AM, and sacrificed 3 hours after the treatment (at 10^{00} AM). Adrenaline treated animals received Adrenaline inj (Demo S.A., Greece) in a dose of 0.5 mg per 100 g body weight in volume of 50 µl. Control animals received saline in the same volume of 50 µl per 100 g body weight. All the animals were sacrificed 3 hours after the treatment and spermatozoa were isolated following the procedure previously described [1].

To monitor the membrane potential of mitochondria (Δψ) in spermatozoa from control and adrenaline treated animals, Tetramethylrhodamine ethyl ester (TMRE) as fluorescent probe was employed according to the procedure published previously [2]. Briefly, after the isolation, $1x10^5$ spermatozoa were loaded in each well of a 96-well black plate, with eight replicates from each animal. Spermatozoa in a 96-well plate were left for 3 hours in the incubator at 37 °C, to recover from the isolation procedure. After that period medium was aspirated, TMRE fluorescent dye was added in a final concentration of 100 nM, and incubated for 20 minutes at 37 °C. Spermatozoa were washed from TMRE fluorescent dye by aspiration and fluorescence was measured in 0,1% BSA-1xPBS solution (Ex/Em 550/590 nm). After the measurement, all wells were washed with 1xPBS solution and frozen at -20 °C until the measurement of protein concentration in each well by Bradford protein assay.

Considering the fact that the lower fluorescent level in this method implies depolarization of the mitochondrial membrane, it was shown that there is a slightly decreased depolarization in adrenaline treated animals compared with control, but not statistically significant (Supplementary figure 1B).

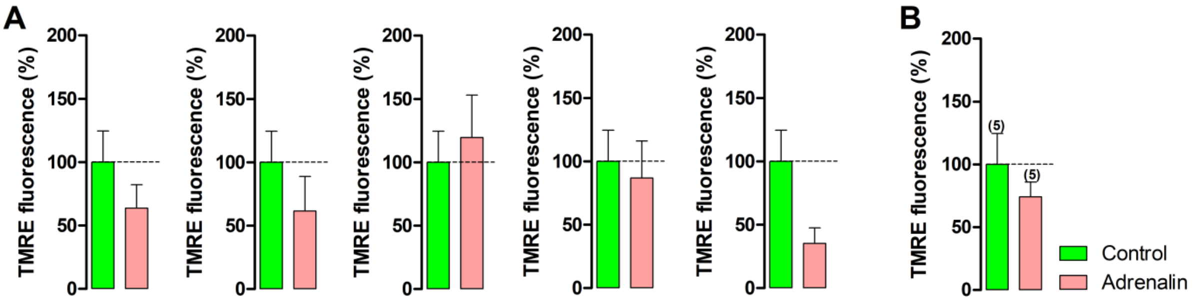

***Supplementary Figure 1*. The relative ratio of TMRE fluorescence (%) in spermatozoa 3 hours after *in vivo* adrenaline treatment of adult rats.**

Membrane potential of mitochondria (Δψ) in spermatozoa from control and adrenaline treated animals was measured using Tetramethylrhodamine ethyl ester (TMRE) as fluorescent probe. TMRE fluorescence (%) is presented as ratio of TMRE fluorescence and protein concentration is each well. For each animal used in the experiment eight replicates was measured. (**A**) TMRE fluorescence (%) of eight replicates for each adrenaline treated animal presented individually, with all the replicates of the control animals. (**B**) TMRE fluorescence (%) of all the adrenaline treated and control animals used in the experiment.

Data bars are mean ± SEM values of one *in vivo* experiment.

SUPPLEMENTARY MATERIALS AND METHODS

MATERIALS - Key resources table

Resource or reagent	Source	Identifier
**Experimental model and biological samples**		
***Wistar* rat**	LaRES and ChronAge Laboratories (DBE, Faculty of Sciences, University of Novi Sad)	http://wwwold.dbe.pmf.uns.ac.rs/en/nauka-eng/lares
**Primary culture of spermatozoa**	Three to four months-old male rats	NA
**Serum**	Three to four months-old male rats	NA
**Commercial Reagents/Assays**		
**Adrenaline research ELISA Kit**	Labor Diagnostika Nord	https://ldn.de
**Corticosterone EIA Kit**	Cayman	https://www.caymanchem.com
**GenElute™ Mammalian Total RNA Miniprep Kit**	Sigma Aldrich	https://www.sigmaaldrich.com
**DNase I (RNase-free) treatment**	New England Biolabs	https://international.neb.com
**High Capacity Kit for cDNA**	Applied Biosystems/Thermo Fisher Scientific	https://www.thermofisher.com
**Power SYBR® Green PCR Master Mix**	Applied Biosystems/Thermo Fisher Scientific	https://www.thermofisher.com
**ATP Bioluminescence CLS II kit**	Roche/ Sigma Aldrich	https://www.sigmaaldrich.com
**Primers**		
**Supplemental tables 1 to 7**	This paper	www.ncbi.nlm.nih.gov/sites/entrez
**Software**		
**GraphPad Prism 5 Software**	GraphPad Prism	https://www.graphpad.com/scientific-software/prism

METHODS

All experiments were carried out in the Laboratory for Reproductive Endocrinology and Signaling and Laboratory for Chronobiology and Aging, Faculty of Sciences at University of Novi Sad (*wwwold.dbe.pmf.uns.ac.rs/en/nauka-eng/lares*). The methods used in this study were carried out in accordance with the relevant guidelines and regulations and were previously reported by our group (for all references please see [1–4]).

Statement of Institutional Review Board
The manuscript is approved by the Committee of the Faculty of Sciences, University of Novi Sad.

Statement that the authors complied with ARRIVE guidelines and Institutional Animal Care and Use Committee guidelines

The authors complied with ARRIVE guidelines and all experiments were in adherence to the ARRIVE guidelines. Besides, all experimental protocols were approved (statement no. 01-201/3) by the local Ethical Committee on Animal Care and Use of the University of Novi Sad operating under the rules of National Council for Animal Welfare and the National Law for Animal Welfare (copyright March 2009), following the NRC publication Guide for the Care and Use of Laboratory Animals and NIH Guide for the Care and Use of Laboratory Animals.

Animals and experimental model of stress

Three-months-old male *Wistar* rats, bred and raised in the accredited Animal Facility of the Faculty of Sciences, University of Novi Sad, Serbia were used for experiments. Animals were raised in controlled environmental conditions (22 ± 2 °C; 14 hours light and 10 hours dark cycle, lights on at 07^{00} h) with food and water *ad libitum*. The experimental model of psychophysical stress by immobilization (IMO) was performed in the morning (from 07^{00} h to 10^{00} h) by the method previously described [1,2,4,5]. Briefly, stressed (3hIMO) rats were bound in a supine position to a wooden board by fixing the rats' limbs using thread, while the head motion was not limited. The animals were divided into the following groups consisting of three to six animals each: control group – unstressed (freely moving) rats; 1x3hIMO – rats subjected to IMO once, for 3 hours; 10x3hIMO – rats subjected to repeat IMO of 3 hours for 10 consecutive days. At the end of IMO period, control and stressed animals were quickly decapitated without anesthesia and trunk blood was collected. Individual serum samples were stored at -70 °C until they were assayed for androgens (testosterone + dihydrotestosterone; T+DHT), adrenaline and corticosterone (CORT) levels. The experiments were repeated three times.

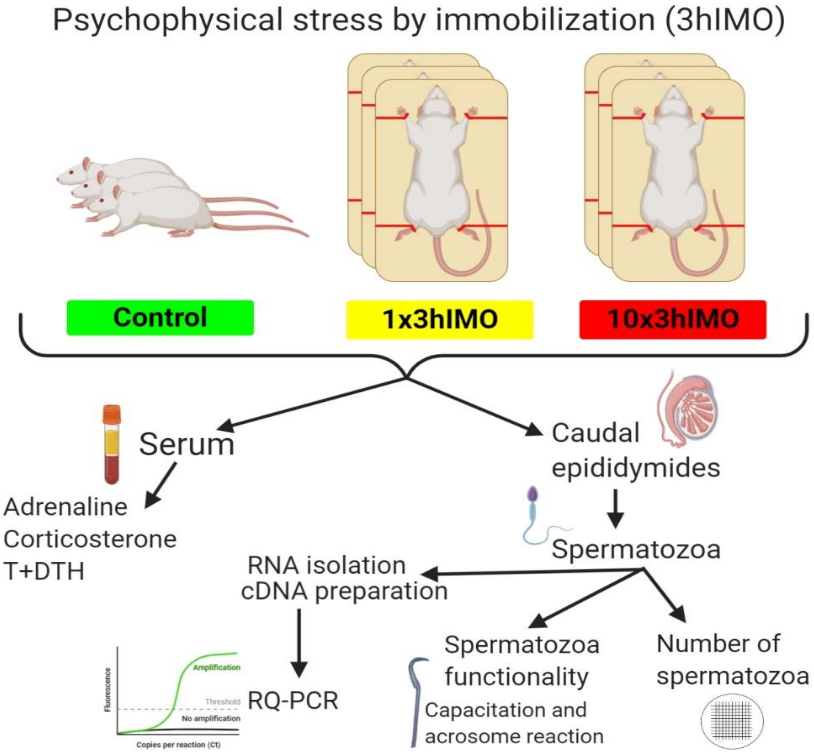

Supplemental scheme 1. **Experimental design used to asses levels of hormones, spermatozoa number and functionality (% acrosome reaction) as well as transcriptional profiles of mitochondrial dynamics markers and related signaling molecules.**

Experimental model of *in vivo* adrenaline treatment

Three-months-old male *Wistar* rats, bred and raised in the accredited Animal Facility of the Faculty of Sciences, University of Novi Sad, Serbia were used for experiments. Animals were raised in controlled environmental conditions (22 ± 2 °C; 14 hours light and 10 hours dark cycle, lights on at 07^{00} AM) with food and water *ad libitum*. Animals were divided into two groups consisting of 5 animals in each group:undisturbed – control group and group of rats treated with adrenaline in concentration similar to that detected in the serum of the repeatedly stressed (10x3hIMO) adult male rats. Adrenaline treated animals received Adrenaline inj solution (Demo S.A., Greece) in a dose of 0.5 mg per 100 g body weight in volume of 50 µl. At the same time, control animals received saline in the same volume of 50 µl per 100 g body weight. All the animals were treated with intramuscular injection at 07^{00} AM, and quickly decapitated 3 hours after the treatment (at 10^{00} AM). After the treatment spermatozoa were isolated from caudal epididymides and used for the measurement of mitochondrial membrane potential ($\Delta\psi$). The Experiment was performed once.

Serum hormones measurement

Level of androgens, in serum, was referred to as T+DHT considering that the anti-testosterone serum №250 showed 100% cross-reactivity with DHT (for references please see [1,2,4]). All samples were measured in duplicate in one assay (sensitivity: 6 pg per tube; intra-assay coefficient of variation 5-8%). Serum adrenaline levels were measured in duplicate, for all samples, using the adrenaline research ELISA Kit (www.ldn.de) with the standard range of 0.45-45 ng/ml and detection limit of 3.9 pg/ml. Serum corticosterone (CORT) levels in all samples were measured in duplicate in one assay by the corticosterone EIA Kit (www.caymanchem.com) with 30 pg/ml as the lowest standard significantly different from blank.

Isolation of spermatozoa and assessment of their functionality (capacitation and acrosome reaction)

Spermatozoa were isolated from caudal epididymides following the WHO laboratory manual (https://www.who.int/reproductivehealth/publications/infertility/9789241547789/en/) with modifications for rat spermatozoa isolation (for reference please see [1]). Briefly, caudal epididymides were quickly removed and the surrounding adipose tissue cut out. Isolated epididymis was placed in a petri dish containing 4 ml of the medium for isolation and preservation of spermatozoa (1% M199 in HBSS with 20 mM HEPES buffer and 5% BSA) or Whitten's Media (100 mM NaCl, 4.7 mM KCl, 1.2 mM KH_2PO_4, 1.2 mM $MgSO_4$, 5.5 mM glucose, 1 mM pyruvic acid and 4.8 mM lactic acid), depending on the subsequent analysis. Epididymides were finely punctuated with 25G needle to enable spermatozoa to be released into the medium and incubated at 37 °C for 10 minutes. Released spermatozoa in the medium were collected, centrifuged 5 minutes at 700*xg* at room temperature, the supernatant was removed and the pellet resuspended in the appropriate medium. Concentrations of isolated spermatozoa were calculated using a Makler counting chamber (Sefi-Medical Instruments, ltd). Isolated spermatozoa were used for the capacitation and acrosome reaction procedure and the rest of spermatozoa were stored at -80 °C, before RNA isolation, and the subsequent gene transcription analysis. **To determine the functionality of the spermatozoa**, approximately $1.5x10^5$ spermatozoa in 50 µl of Whitten's Media were mixed with 350 µl WH+ media (Whitten's Media supplemented with the 10 mg/ml BSA

(Bovine Serum Albumin) and 20 mM of $NaHCO_3$, to stimulate the capacitation) with a drop of mineral oil, for 1 hour at 37 °C (5% CO_2). Capacitated spermatozoa, in the volume of 50 µl, were transferred in two new tubes, one without the progesterone and one with 15 µM progesterone (PROG), with the drop of mineral oil, and incubated for 30 minutes at 37 °C (5% CO_2). Progesterone was added to activate the acrosome reaction, where tubes without PROG were present as the control of the acrosome reaction. After the acrosome reaction, 20 µl of the spermatozoa suspension from each tube were mixed with 100 µl of the fixation solution (20 mM Na_2HPO_4, 150 mM NaCl and 7.5% formaldehyde) and incubated for 20 minutes at room temperature. Subsequently, spermatozoa were centrifuged for 1 minute at 12000xg. The supernatant was discarded and spermatozoa washed with 100 mM ammonium acetate, pH 9. Smears of fixed spermatozoa were prepared on microscopic slides, air-dried and stained by covering the slides with staining solution (0.04% Coomassie Blue – G250, 50% methanol and 10% acetic acid) for 5 minutes at room temperature, rinsed with distilled water and allowed to air-dry. Stained smears were analyzed using the microscope Leica DMLB 100T (Leica, Wetzlar, Germany), with 1000x magnification. Ten to fifteen photos per slide were taken by Leica MC190 camera (Leica, Wetzlar, Germany) and LAS Ver 4.8.0 software, and up to 100 spermatozoa per slide counted to determine the acrosomal status. Blue staining in the acrosomal region of the head indicated intact acrosome, whereas spermatozoa without blue staining in the acrosomal region were considered acrosome-reacted. Data are presented as the percentage of acrosome-reacted spermatozoa ± SEM.

Measurement of ATP level

The ATP level was determined using ATP Bioluminescence CLS II kit following the manufacturer's instructions (*www.sigmaaldrich.com*) Spermatozoa (1×10^6) were lysed in distilled H_2O and mixed with Tris-EDTA in ratio of 1:9 (v/v). Lysed spermatozoa in Tris-EDTA were incubated in water bath (100 °C/3 min), centrifuged for 1 minute at 900×g. Supernatant was used for the measurement of ATP while the pellet was further used for measurement of protein concentration using Bradford protein assay. Sample and standard were mixed with Luciferase reagent in ratio of 1:1 (v/v), and luminescence was measured by the Biosystems/luminometer (Fluoroscan, Ascent, FL), as described previously by our group (for references please see [6]).

Mitochondrial membrane potential (Δψ) measurement using TMRE fluorescent dye

To monitor the membrane potential of mitochondria (Δψ) in spermatozoa from control and adrenaline treated animals, Tetramethylrhodamine ethyl ester (TMRE) as fluorescent probe was employed according to the procedure published previously [2]. Briefly, after the isolation, $1x10^5$ spermatozoa were loaded in each well of a 96-well black plate, with eight replicates from each animal. Spermatozoa in the 96-well plate were left for 3 hours in the incubator at 37 °C, to recover from the isolation procedure. After that period medium was aspirated, TMRE fluorescent dye was added in a final concentration of 100 nM, and incubated for 20 minutes at 37 °C. Spermatozoa were washed from TMRE fluorescent dye by aspiration and fluorescence was measured in 0,1% BSA-1xPBS solution (Ex/Em 550/590 nm). After the measurement, all wells were washed with 1xPBS solution and frozen at -20 °C until the measurement of protein concentration in each well by Bradford protein assay.

RNA isolation and cDNA synthesis

Isolation of total RNA, from spermatozoa samples, was done using the GenElute™ Mammalian Total RNA Miniprep Kit according to the protocol recommended by the manufacturer (www.sigmaaldrich.com), following the DNase I (RNase-free) treatment according to the manufacturer's protocol (www.neb.com). First-strand cDNA was synthesized using the High Capacity Kit for cDNA preparation according to the manufacturer's instructions (www.thermofisher.com). Negative controls consisting of non-reverse transcribed samples were included in each set of reactions. Quality of RNA and DNA integrity was checked using control primers for *Gapdh*, as described previously by our group (for references please see [1,2,4]).

Relative quantification of gene expression

The quantification of relative gene expression was done by real-time PCR (RQ-PCR) using SYBR®Green-based chemistry from Applied Biosystems (www.thermofisher.com) in the presence of 10 ng of cDNA (calculated from starting RNA) in an aliquot of 2.5 µl, and specific primers at the final concentration of 500 nM. Primer sequences used for real-time PCR analysis and Ct values, as well as GenBank accession codes for full gene sequences (www.ncbi.nlm.nih.gov/sites/entrez), are given in *Supplemental tables 1 to 7*. The transcription of *Gapdh* was measured in the same samples and used to correct the variations in cDNA content between samples. Relative quantification of each gene was performed in duplicate, three times for each sample of three independent *in vivo* experiments. The real-time PCR reactions were carried out in the Eppendorf Master Cycler ep RealPlex 4 and post-run analyzes were performed using Mastercycler® eprealplex Software (for references please see (for references please see [1,2,4]).

Supplemental table 1. **Primers sequences used for the real-time PCR analysis of molecular markers of mitochondrial biogenesis.**

Gene	Accession code	Primers	Primer length	Product length	Ct value
Ppargc1a	NM_031347	F: 5'-AGCCGTAGGCCCAGGTATGACA-3'<br>R: 5'-TGCTTGGCCCTTTCAGACTCCC-3'	22 bp<br>22 bp	107 bp	30.54
Ppargc1b	NM_176075	F: 5'-ACCTTCCGGTGTTCGGAGCATG-3'<br>R: 5'-GTGGAAGGAGGGCTCATTGCGT-3'	22 bp<br>22 bp	81 bp	28.71
Tfam	NM_031326	F: 5'-TATAGTCGTCGGCCCGAGGGAT-3'<br>R: 5'-AAGGCTGACAGGCGAGGGTATG-3'	22 bp<br>22 bp	125 bp	28.13
Nrf1	NM_001100708	F: 5'-GACCATCCAGACGACGCAAGCA-3'<br>R: 5'-ATGGGCGGCAGCTTCACTGTT-3'	22 bp<br>21 bp	136 bp	27.89
Nrf2a	NM_001108841	F: 5'-AGCGGAACTGAACCGCTTGGT-3'<br>R: 5'-GTGACTGGCTGAGCAATCCCGT-3'	21 bp<br>22 bp	84 bp	27.59
Ppara	NM_013196	F: 5'-GTCCTGGAACTGAAGCGACGCT-3'<br>R: 5'-TTACGCCCAAATGCACCACGC-3'	22 bp<br>21 bp	110 bp	28.71
Ppard	NM_013141	F: 5'-ACGGTAAAGGCGGTCCATCTGC-3'<br>R: 5'-TCCTCCTGTGGCTGTTCCATGAC-3'	22 bp<br>23 bp	109 bp	26.38
mtNd1		F: 5' GCGTGGGAGGAGCATCAGGG 3'<br>R: 5' GCGAATGGTCCTGCGGCGT A 3'	20 bp<br>20 bp	271 bp	18.20
Gapdh	NM_017008	F: 5'-TGCCAAGTATGATGACATCAAGAAG-3'<br>R: 5'-AGCCCAGGATGCCCTTTAGT-3'	25 bp<br>20 bp	110 bp	20.72

Primers were designed by using software Primer Express 3.0 (Applied Biosystems) and full genes sequences from the NCBI Entrez Nucleotide database (www.ncbi.nlm.nih.gov/sites/entrez). F - forward; R - reverse.

Supplemental table 2. **Primers sequences used for the real-time PCR analysis of molecular markers of mitochondrial fusion and architecture.**

Gene	Accession code	Primers	Primer length	Product length	Ct value
Mfn1	NM_138976.1	F: 5'-CCTTGTACATCGATTCCTGGGTTC-3'<br>R: 5'-CCTGGGCTGCATTATCTGGTG-3'	24 bp<br>21 bp	143 bp	28.91
Mfn2	NM_130894.4	F: 5'-TCAAGCGCCAGTTTGTGGAG-3'<br>R: 5'-CACAGATGAGCAAATGTCCCAGA-3'	20 bp<br>23 bp	118 bp	27.18
Opa1	NM_133585.3	F: 5'-AAAAGCCCTTCCCAGTTCAGA-3'<br>R: 5'-TACCCGCAGTGAAGAAATCCTT-3'	21 bp<br>22 bp	101 bp	26.18
Gapdh	NM_017008	F: 5'-TGCCAAGTATGATGACATCAAGAAG-3'<br>R: 5'-AGCCCAGGATGCCCTTTAGT-3'	25 bp<br>20 bp	110 bp	20.69

Primers were designed by using software Primer Express 3.0 (Applied Biosystems) and full genes sequences from NCBI Entrez Nucleotide database (*www.ncbi.nlm.nih.gov/sites/entrez*). F - forward; R - reverse.

Supplemental table 3. **Primers sequences used for the real-time PCR analysis of molecular markers of mitochondrial fission.**

Gene	Accession code	Primers	Primer length	Product length	Ct value
Fis1	NM_001105919.1	F: 5'-ACGCCTGCCGTTACTTCTTC-3'<br>R: 5'-GCAACCCTGCAATCCTTCAC-3'	20 bp<br>20 bp	108 bp	28.89
Drp1	NM_053655.3	F: 5'-AGGTTGCCCGTGACAAATGA-3'<br>R: 5'-CACAGGCATCAGCAAAGTCG-3'	20 bp<br>20 bp	94 bp	28.85
Gapdh	NM_017008	F: 5'-TGCCAAGTATGATGACATCAAGAAG-3'<br>R: 5'-AGCCCAGGATGCCCTTTAGT-3'	25 bp<br>20 bp	110 bp	22.44

Primers were designed by using software Primer Express 3.0 (Applied Biosystems) and full genes sequences from NCBI Entrez Nucleotide database (*www.ncbi.nlm.nih.gov/sites/entrez*). F - forward; R - reverse.

Supplemental table 4. **Primers sequences used for the real-time PCR analysis of molecular markers of mitochondrial autophagy.**

Gene	Accession code	Primers	Primer length	Product length	Ct value
Pink1	NM_001106694.1	F: 5'-CAAGCAAGTGTCTGACCCAC-3'<br>R: 5'-GCTTCATACACAGCGGCATT-3'	20 bp<br>20 bp	111 bp	26.23
Prkn	NM_020093.1	F: 5'-CTTCCAGCTCAAGGAAGTGG-3'<br>R: 5'-CAGAGGCATTTGTTTCGTGA-3'	20 bp<br>20 bp	182 bp	32.47
Tfeb	NM_001025707.1	F: 5'-CGACAACATTATGCGCCTGG-3'<br>R: 5'-CTGTACACGTTCAGGTGGCT-3'	20 bp<br>20 bp	102 bp	28.53
Gapdh	NM_017008	F: 5'-TGCCAAGTATGATGACATCAAGAAG-3'<br>R: 5'-AGCCCAGGATGCCCTTTAGT-3'	25 bp<br>20 bp	110 bp	21.45

Primers were designed by using software Primer Express 3.0 (Applied Biosystems) and full genes sequences from NCBI Entrez Nucleotide database (*www.ncbi.nlm.nih.gov/sites/entrez*). F - forward; R - reverse.

***Supplemental table 5.* Primers sequences used for the real-time PCR analysis of molecular markers of mitochondrial functionality.**

Gene	Accession code	Primers	Primer length	Product length	Ct value
Cox4i1	NM_017202	F: 5'-CGCTGAGATGAACAAGGGCACC-3'<br>R: 5'-TCCCAGATCAGCACAAGCGCA-3'	22 bp<br>21 bp	93 bp	22.15
Cox4i2	NM_053472	F: 5'-CACAGCCCAGGAAGTGCTGCTA-3'<br>R: 5'-TGTGCAGTAAGGCTCATCCGGC-3'	22 bp<br>22 bp	105 bp	31.39
Cytc	NM_012839	F:5'-GCAAGCATAAGACTGGACCAAA-3'<br>R:5'-TTGTTGGCATCTGTGTAAGAGAATC-3'	22 bp<br>25 bp	88 bp	23.52
Ucp1	NM_012682	F: 5'-TCAGCTCTTGTCGCCGGGTTT-3'<br>R: 5'-TGCACAGCTGGGTACACTTGGG-3'	21 bp<br>22 bp	114 bp	29.73
Ucp2	NM_019354	F: 5'-ACGACCTCCCTTGCCACTTCAC-3'<br>R: 5'-GGTACTGGCCCAAGGCAGAGTT-3'	22 bp<br>22 bp	117 bp	22.65
Ucp3	NM_013167	F: 5'-TGCTCAACCCACGGATGTGGT-3'<br>R: 5'-CCTGGCGATGGTTCTGTAGGCA-3'	21 bp<br>22 bp	112 bp	29.24
Gapdh	NM_017008	F: 5'-TGCCAAGTATGATGACATCAAGAAG-3'<br>R: 5'-AGCCCAGGATGCCCTTTAGT-3'	25 bp<br>20 bp	110 bp	21.01

Primers were designed by using software Primer Express 3.0 (Applied Biosystems) and full genes sequences from the NCBI Entrez Nucleotide database (*www.ncbi.nlm.nih.gov/sites/entrez*). F - forward; R - reverse.

***Supplemental table 6.* Primers sequences used for the real-time PCR analysis of cAMP signaling elements.**

Gene	Accession code	Primers	Primer length	Product length	Ct value
Adcy3	NM_130779	F: 5'-GCATCGAAACCTACCTCATCA-3'<br>R: 5'-TGGGCTCCTTGGTCTCA ATAA-3'	21 bp<br>21 bp	141 bp	31.87
Adcy5	NM_022600	F: 5'-AACCAGGTGAACGCATGTCA-3'<br>R: 5'-CTCTGGGAAGTTGCAGTTGGA-3'	20 bp<br>21 bp	105 bp	30.13
Adcy6	NM_012821	F: 5'-CTGCCTCAGCCTGCTTATGTG-3'<br>R: 5'-GGAGTCCTGGCGGAAGCT-3'	21 bp<br>18 bp	99 bp	27.46
Adcy7	NM_053396	F: 5'-TTCCGTGCGTGTAACCCGCT-3'<br>R: 5'-GCCTTCTGCCTCCGTCCGTT-3'	20 bp<br>20 bp	123 bp	27.54
Adcy8	NM_017142	F: 5'-ATTGCCTCAGTGGTGACTA-3'<br>R: 5'-CAAACTCTCCTCGGGCT-3'	19 bp<br>17 bp	113 bp	29.21
Adcy9	NM_001106980	F: 5'-TCACCAAGCTGTACGCCCGG-3'<br>R: 5'-GGGCTGTCAACACGTCCCGA-3'	20 bp<br>20 bp	124 bp	30.45
Adcy10	NM_021684	F: 5'-CCAGGCATCGTGACCTGCGA-3'<br>R: 5'-ACTGGTCCGGGATCCGCAAC-3'	20 bp<br>20 bp	113 bp	30.83
Prkaca	NM_001100922.1	F: 5'-TCAGTGAGCCCCACGCCCGTT-3'<br>R: 5'-TCTCGGGCTTCAGGTCCCGG-3'	21 bp<br>20 bp	99 bp	27.51
Prkacb	NM_001077645	F: 5'-GGGTCATGGGGAACACGGCG-3'<br>R: 5'-CCAGCATTACTCGGGGGAGGGT-3'	20 bp<br>22 bp	124 bp	28.76
Prkar1a	NM_013181	F: 5'-TGTGCTGCAGCGTCGGTCAG-3'<br>R: 5'-AGTGGCAGCCCGAGGACGAT-3'	20 bp<br>20 bp	112 bp	25.94
Prkar2a	NM_019264	F: 5'-GCCCGACCTCGTCGACTTCG-3'<br>R: 5'-TCCTGCGCGTGAAAGGTCGT-3'	20 bp<br>20 bp	108 bp	27.71
Prkar2b	NM_001030020	F: 5'-CCCATGCGCTCCGATTCCGA-3'<br>R:5'-GCACATACCGAGGCACGCCT-3'	20 bp<br>20 bp	107 bp	31.51
Gapdh	NM_017008	F: 5'-TGCCAAGTATGATGACATCAAGAAG-3'<br>R: 5'-AGCCCAGGATGCCCTTTAGT-3'	25 bp<br>20 bp	110 bp	21.85

Primers were designed by using software Primer Express 3.0 (Applied Biosystems) and full genes sequences from NCBI Entrez Nucleotide database (*www.ncbi.nlm.nih.gov/sites/entrez*). F - forward; R - reverse.

Supplemental table 7. **Primers sequences used for the real-time PCR analysis of MAPK signaling elements.**

Gene	Accession code	Primers	Primer length	Product length	Ct value
Mapk1	NM_053842.1	F: 5'-GTTCTGCACCGTGACCTCAAG-3'<br>R: 5'-GCAAGGCCAAAGTCACAGATC-3'	21 bp<br>21 bp	80 bp	27.98
Mapk3	NM_017347.2	F: 5'-TCCCTCTCAAGCTGCCACAT-3'<br>R: 5'-ACATCCAATCACCCACACACA-3'	20 bp<br>21 bp	60 bp	27.56
Mapk6	NM_031622.2	F: 5'-CATTTGAACTGGCATGTCGTTT-3'<br>R: 5'-CCTGCACTGCATTGTTTTGC-3'	22 bp<br>20 bp	62 bp	27.80
Mapk7	NM_001191547.1	F: 5'-GCCCCTTCCACTAGCCTTTT-3'<br>R: 5'-GAACCAGGCAACCCACTAGGT-3'	20 bp<br>21 bp	62 bp	28.83
Mapk8	NM_053829.2	F: 5'-TCAACGTCTGGTATGATCCTTCA-3'<br>R: 5'- CTGCTTGTCAGGGATCTTTGG-3'	23 bp<br>21 bp	62 bp	29.11
Mapk9	NM_017322.1<br>NM_001270544.1<br>NM_001270545.1	F: 5'-GGAAGGCTGCCGATGAAA-3'<br>R: 5'-AGCCAGAGTCCTTCACAGACAAG-3'	18 bp<br>23 bp	57 bp	28.65
Mapk11	NM_001109532.2	F: 5'-GGGCGCTGACCTGAATAACA-3'<br>R: 5'-GCAGCAGCTGGTAGACAAGGA-3'	20 bp<br>21 bp	80 bp	30.43
Mapk12	NM_021746.1	F: 5′-GGATGTGTTCACTCCCGATGA-3′<br>R: 5′-CCAGGTCAGTGCCCATGAAT-3′	21 bp<br>20 bp	80 bp	28.89
Mapk13	NM_019231.2	F: 5′-CTGGTCTGTTGGCTGCATCA-3′<br>R: 5′-TCAGCTGGTCCAGGTAGTCCTT-3′	20 bp<br>22 bp	80 bp	28.28
Mapk14	NM_031020.2	F: 5′-GCTGTCGACCTGCTGGAAAA-3′<br>R: 5′- TAGGCATGCGCAAGAGCTT-3′	20 bp<br>19 bp	80 bp	27.19
Gapdh	NM_017008	F: 5'-TGCCAAGTATGATGACATCAAGAAG-3'<br>R: 5'-AGCCCAGGATGCCCTTTAGT-3'	25 bp<br>20 bp	110 bp	21.93

Primers were designed by using software Primer Express 3.0 (Applied Biosystems) and full genes sequences from NCBI Entrez Nucleotide database (*www.ncbi.nlm.nih.gov/sites/entrez*). F - forward; R - reverse.

Statistical analysis

Results of the experiments represent group means ± SEM values of the individual variation from three independent experiments (3 to 6 rats per group). Results from each experiment were analyzed by Mann-Whitney's unpaired nonparametric two-tailed test (for two-point data experiments), or by one-way ANOVA for group comparison, followed by Student-Newman-Keuls multiple range test. All the statistical analyses were done using GraphPad Prism 5 Software (GraphPad Software 287 Inc., La Jolla, CA, USA). In all cases, p-value <0.05 was considered to be statistically significant.

References

1. Starovlah, I.M.; Radovic Pletikosic, S.M.; Kostic, T.S.; Andric, S.A. Reduced Spermatozoa Functionality during Stress Is the Consequence of Adrenergic-Mediated Disturbance of Mitochondrial Dynamics Markers. *Sci. Rep.* **2020**, *10*, 16813, doi:10.1038/s41598-020-73630-y.
2. Gak, I.A.; Radovic, S.M.; Dukic, A.R.; Janjic, M.M.; Stojkov-Mimic, N.J.; Kostic, T.S.; Andric, S.A. Stress Triggers Mitochondrial Biogenesis to Preserve Steroidogenesis in Leydig Cells. *Biochim. Biophys. Acta BBA - Mol. Cell Res.* **2015**, *1853*, 2217–2227, doi:10.1016/j.bbamcr.2015.05.030.
3. Radovic Pletikosic, S.M.; Starovlah, I.M.; Miljkovic, D.; Bajic, D.M.; Capo, I.; Nef, S.; Kostic, T.S.; Andric, S.A. Deficiency in Insulin-like Growth Factors Signalling in Mouse Leydig Cells Increase Conversion of Testosterone to Estradiol Because of Feminization. *Acta Physiol.* **2020**, *00*, e13563, doi:10.1111/apha.13563.

4. Starovlah, I.M.; Sava, R.M.; Marinovic, M.A.; Kostic, T.S.; Andric, S.A. Psychophysical Stress Disturbs Expression of Mitochondrial Biogenesis Markers in Hypothalamus and Adenohypophysis. *Biol. Serbica* **2017**, *39*, 43–51.
5. Kvetňanský, R.; Weise, V.K.; Kopin, I.J. Elevation of Adrenal Tyrosine Hydroxylase and Phenylethanolamine-N-Methyl Transferase by Repeated Immobilization of Rats. *Endocrinology* **1970**, *87*, 744–749, doi:10.1210/endo-87-4-744.
6. Medar, M.L.J.; Marinkovic, D.Z.; Kojic, Z.; Becin, A.P.; Starovlah, I.M.; Kravic-Stevovic, T.; Andric, S.A.; Kostic, T.S. Dependence of Leydig Cell's Mitochondrial Physiology on Luteinizing Hormone Signaling. *Life* **2021**, *11*, 19, doi:10.3390/life11010019.
